# An intelligent telemonitoring application for coronavirus patients: reCOVeryaID

**DOI:** 10.3389/fdata.2023.1205766

**Published:** 2023-09-18

**Authors:** Daniela D'Auria, Raffaele Russo, Alfonso Fedele, Federica Addabbo, Diego Calvanese

**Affiliations:** ^1^Faculty of Engineering, Free University of Bozen-Bolzano, Bolzano, Italy; ^2^Pineta Grande Hospital, Caserta, Italy; ^3^University Riuniti Hospital, Ancona, Italy; ^4^Kronosan Srl, Montevergine Hospital, Mercogliano, Italy; ^5^Department of Computing Science, Umeå University, Umeå, Sweden

**Keywords:** artificial intelligence, coronavirus, COVID-19, eHealth, long-term monitoring, rule-based system, telehealth, telemedicine

## Abstract

The COVID-19 emergency underscored the importance of resolving crucial issues of territorial health monitoring, such as overloaded phone lines, doctors exposed to infection, chronically ill patients unable to access hospitals, etc. In fact, it often happened that people would call doctors/hospitals just out of anxiety, not realizing that they were clogging up communications, thus causing problems for those who needed them most; such people, often elderly, have often felt lonely and abandoned by the health care system because of poor telemedicine. In addition, doctors were unable to follow up on the most serious cases or make sure that others did not worsen. Thus, uring the first pandemic wave we had the idea to design a system that could help people alleviate their fears and be constantly monitored by doctors both in hospitals and at home; consequently, we developed reCOVeryaID, a telemonitoring application for coronavirus patients. It is an autonomous application supported by a knowledge base that can react promptly and inform medical doctors if dangerous trends in the patient's short- and long-term vital signs are detected. In this paper, we also validate the knowledge-base rules in real-world settings by testing them on data from real patients infected with COVID-19.

## 1. Introduction

COVID-19 is an *infectious respiratory disease* caused by the virus called SARS-CoV-2, which belongs to the coronavirus family. In the course of the disease, after an initial phase with a flu-like course, a very severe respiratory syndrome may occur, related to the development of bilateral interstitial pneumonia. Its symptoms involve difficulty breathing, dyspnea, breathlessness and increased heart rate. In fact, COVID-19 pneumonia leads to a decrease in blood oxygen level (saturation) without the patient realizing it, until the urgency of hospitalization arises. Therefore, it is necessary to monitor individuals who are under observation for COVID-19 infection at home in order to check the saturation level so that it does not fall below the established threshold, especially in the absence of previous diseases affecting the respiratory system. In such situations, by trending the saturation data, the medical staff will be able to tell whether or not that patient, asymptomatic, symptomatic, or pre-symptomatic and in home isolation, should be hospitalized, thus arriving at an *early hospitalization* before the clinical picture may worsen. The development of an intelligent telemonitoring system, involving the use of traditional diagnostic devices such as a thermometer and oximeter, will thus make it possible:

1. Leave individuals who are not in imminent danger at home, thus avoiding occupying hospital beds;
2. Monitor individuals at risk with possible respiratory crisis;
3. Monitor the part of the population that has not been tested for COVID-19 but may be asymptomatic or pre-symptomatic (such as patients in precautionary quarantine);
4. Measure essential parameters in order to avoid a respiratory crisis for patients with COVID-19 in non-severe form;
5. Allow General Practitioners to keep the patient under constant supervision, thus avoiding the risk of possible infections due to direct and repeated contact over time.

In order to achieve these goals, we designed and developed *reCOVeryaID*, an intelligent telemonitoring application for symptomatic, asymptomatic, and pre-symptomatic coronavirus patients, which we describe in this paper.

The remainder of the paper is organized as follows. In the next section, we show the related work in the eHealth context, with particular focus on monitoring vital signs, and in Section 3 we give an overview of the *reCOVeryaID* prototype, also focusing on the communication protocol between patient and medical doctor. Then, Section 4 illustrates in detail the knowledge-base rules of the framework, aimed at promptly detecting short-term (Section 4.1) and long-term alerts (Section 4.2). Additionally, Section 5 shows the experimental results, and Section 6 concludes the paper and outlines future work.

## 2. Related work

From the pandemic situation we have just experienced, and what has happened since the pandemic began, it is clear that in general, telemedicine in Italy and the rest of the world, never really got started. Among other benefits, apart from the clinical one, it could have saved a lot of money for several health systems, and from the very beginning of the pandemic it could have been very useful in making the monitoring work of primary care physicians on COVID-19 patients (and of course, not only on them) more reliable and lighter (Charles, 2000). The potential of telemedicine, on the other hand, is enormous (Ali and Khoja, 2020; Chauhan et al., 2020; Hollander and Carr, 2020; Monaghesh and Hajizadeh, 2020). This is evidenced by the piecemeal trials underway in some places around the world, as well as the emergence of startups, devices and useful technologies for remote monitoring, but also the interest of health insurance companies that now offer teleconsultations and much more refined solutions in their fee-for-service packages; from wearable devices to virtual triage, from vital signs monitoring to remote examinations (at least those that are feasible), from which the various national health systems could also benefit.

The alarm in this regard, or rather the push for the use of telemedicine systems, has also recently come from the various Rheumatology Societies, as patients with lupus, rheumatoid arthritis, vasculitis, and other similar chronic and often autoimmune diseases need timely diagnosis and especially appropriate therapeutic management, as well as constant monitoring (Danhieux et al., 2020; Dimitroulas and Bertsias., 2020; Mason et al., 2020; Wright et al., 2020; Coupet et al., 2021; Hacker et al., 2021). In fact, these companies are proposing to invest heavily in telemedicine, and some of them have already developed online platforms dedicated to rheumatology patients, but these only work in certain territories. A similar model should be structured and implemented everywhere, for all diseases.

Considering that, for example, in Italy, where we had one of the largest pandemic spikes in the world at the beginning of the first pandemic wave, even electronic medical records, a relatively simple tool for coordinating all the services provided to citizens, never took off, we realize that the road to digitizing public health is still a long one. Despite the mandatory acceleration of the pandemic, so far only dematerialized prescriptions have found widespread use, and this mostly reduces telemedicine to the (often busy) telephone of General Practitioners.

In some cases, attempts have been made to buffer the lack of adequate telemedicine systems with the help of AI to make predictions about disease or diagnosis (Li et al., 2020). The use of artificial intelligence has been proposed to analyze CT scans of the chest (Han et al., 2020) and make early diagnosis of COVID-19, as well as to detect predictive criteria for cytokine storm (Caricchio et al., 2021) or assess specific laboratory parameters (such as lymphocyte count or hsCRP) to predict the worsening clinical condition of the patient. Apart from the difficulty of validating new valid parameters, the main problem with this work is that patients have to perform invasive “screening” procedures, such as CT scans or blood draws involving hospitals and laboratories, thus making the diagnostic process costly. For this reason, other authors propose algorithms to predict the risk of death in hospitalized patients (Gao et al., 2020) and the need for invasive ventilation (Burdick et al., 2020), acting only when it is too late and the patient already needs hospitalization.

Considering the above, and consistent with past findings from various studies on the importance of monitoring saturation level through the use of an oximeter (Taguchi et al., 1994; Solé et al., 2009; Scott and McDougall, 2017; Elliott and Baird, 2019; Takei et al., 2020), there is a clear need to monitor individuals who are home under observation for COVID-19 infection to check that the saturation level does not fall below a threshold, especially in the absence of previous respiratory illness. In such situations, by trending saturation data, medical staff will be able to understand whether that patient, asymptomatic, symptomatic or pre-symptomatic and in home isolation, should be admitted or not, thus arriving at an early admission before the clinical picture may worsen.

Consequently, an integrated home care/telemedicine system involving the use of a saturation device and a thermometer can address all the problems mentioned: The patient could provide the data needed for analysis on his or her own, while an algorithm processes and filters the results, presenting any alerts to the referring medical doctor in real time. The advantages will be that the patient will not have to undergo invasive examinations, such as taking CT scans or blood samples, and that there will be less hospital attendance in case of mild symptoms that do not require intensive treatment.

Other recent and relevant telemonitoring systems based on vital parameter monitoring are listed below. Specifically, Wiffen et al. (2023) support the refinement of data collection and processing toward the development of a robust app that is suitable for routine clinical use, whereas Ko et al. (2023) conducted in-person home visits at least once a day, with nursing visits up to 3 times a day for intravenous therapy; additionally, patients were discharged from the program when they met conventional inpatient discharge criteria. Murali (2023) offer a more effective way to keep track of the patient's medical system. They mainly focus on using Machine Learning (ML), Internet of Things (IoT), and cloud services for patient monitoring. In addition, their system can be used as a sophisticated IOT-equipped real-time patient monitoring system to track the patient's vital signs, such as body temperature, blood pressure, heart rate, and oxygen saturation. Then, Totuk et al. (2023) compared measurements of Value Stream Mapping (VSM) with pulse oximetry probes, smartphones, and Blood Group Antigens (BGA). The Integrated Comprehensive Care (ICC) of the oxygen saturation of arterial blood (SaO2) measurements performed by the VSM, smartphone, and BGA indicated an excellent agreement between the devices. Similarly, the ICC value of the Heart Rate (HR) measurements showed an excellent agreement between the VSM and smartphone. Furthermore, Smith et al. (2021) claim that the future success of wearable technologies lies in establishing clinical confidence in the quality of the data measured and the accurate interpretation of that data in the context of the individual, the environment, and the activity performed; in fact, in the near future wearable physiological monitoring could improve point-of-care diagnostic accuracy and inform critical command and medical decisions.

To sum up, another strength of the *reCOVeryaID* prototype is that it is flexible and general enough that it can also be integrated with other systems dealing with different diseases; in fact, it could be integrated with Italian clinical notes (D'Auria et al., 2023), telemonitoring systems based on intelligent agents and complex event processing (Persia et al., 2021; De Lauretis et al., 2023), and a multi-agent based system for epilepsy detection and prediction in neuropediatrics (Bertoncelli et al., 2023).

## 3. The reCOVeryaID prototype

To meet the above requirements, we have developed an intelligent telemonitoring application.^1^ Specifically, we exploited the following technologies:

- *Flutter*^2^: Google's open source framework for creating cross-platform (Android, iOS, Web, Linux, Windows, MacOS, Embedded) applications compiled natively from a single code base;
- *Dart*^3^: open source language supported by Google and optimized to have fast applications on any kind of platform (Android, iOS, Web, Linux, Windows, MacOS, Embedded);

and the following tools:

- An oximeter to measure oxygen saturation (SpO2), which can be used by the patient at home;
- A thermometer for measuring body temperature, which can be used by the patient at home;
- The patient's smartphone (or PC);
- The doctor's smartphone (or PC); and
- The free reCOVeryaID Web-App.

Specifically, the application provides for the monitoring of patients by general practitioners or other medical specialists based on measurements sent by the patient through the application. Each measurement must contain the *body temperature value*, the *SpO2 value*, the *heart rate (HR)*, and the *related timestamp*. The system, using rules stored in a specific knowledge base, will assign an alert level (*red, yellow*, or *green*) to each measurement. To design the rules to be adopted to generate alerts on the measurements taken by the patient, it was decided to use *threshold values* of temperature, SpO2 and HR that have been validated by medical experts in the field. At this point, once the medical doctor has received the measurement and its alert level and viewed (easily via the Web-App) the historical trend of the various measurements, (s)he can easily respond to the patient with an appropriate feedback message. This message will be:

- An OK, in case of a green alert;
- A specific request to the patient, in case of a yellow alert;
- A notification of intervention by an ambulance, in case of a red alert.

In the last case, the application will call an ambulance, subject to confirmation by the doctor. In addition, the system will periodically perform more detailed statistical analyses of the latest *N* measurements of each individual patient. These checks will be stored in a database, which will keep track of *patient ID, measurement interval*, and *outcome*, and may generate additional alerts no longer tied to the last timestamp, but to a larger time interval. If, for example, *M* measurements out of the last *N* (with *M* ≤ *N*) are too close to the red threshold of one of the three vital parameters, the system will signal a *long-term anomaly* of the affected parameter. *reCOVeryaID* is currently a prototype. The features that make this system innovative are the quick and easy interaction protocol between the patient and the medical doctor, as well as the original system of rules—involving vital parameters—stored in the knowledge base aimed at generating alerts. Taking these peculiarities into account, *reCOVeryaID* is easily transferable to other fields of application not strictly related to COVID-19 emergency, such as monitoring patients with diseases, such as diabetes or hypertension, which broaden its potential and strengthen its acceptance by medical doctors.

### 3.1. The communication protocol

**Figure 1:**
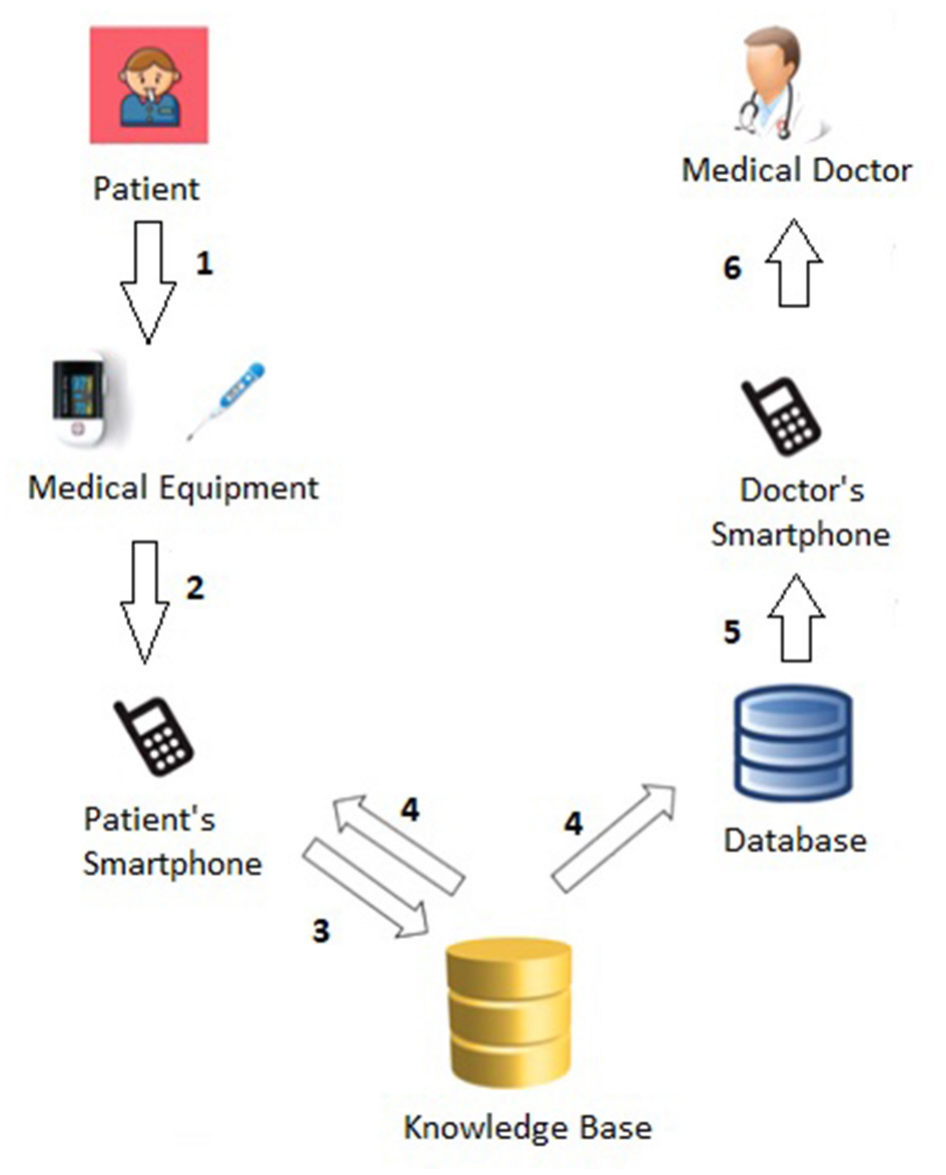
*Patient*→*Medical Doctor* communication protocol.

Figure 1 shows more details about the *Patient*→*Medical Doctor Communication Protocol*; specifically, it consists of the following steps:

1. The patient (periodically) monitors his or her vital parameters through the medical equipment, namely the *oximeter* and the *thermometer*;
2. The current measurement (of *temperature, oxygen saturation*, and *heart rate*) is forwarded to the patient's smartphone either manually or automatically via bluetooth.^4^.
3. The knowledge base (see Section 4) analyzes the current measurement (as well as the last *N* ones) and computes the related *short-term* (Section 4.1) and *long-term* (Section 4.2) alerts.
4. The measurement and related short- and long-term alerts are immediately shown to the patient and stored in the database.
5. The computed alerts are forwarded to the doctor's smartphone.
6. The medical doctor has access to all alerts, sorted according to the level of urgency (i.e., from *red* to *green*).

**Figure 2:**
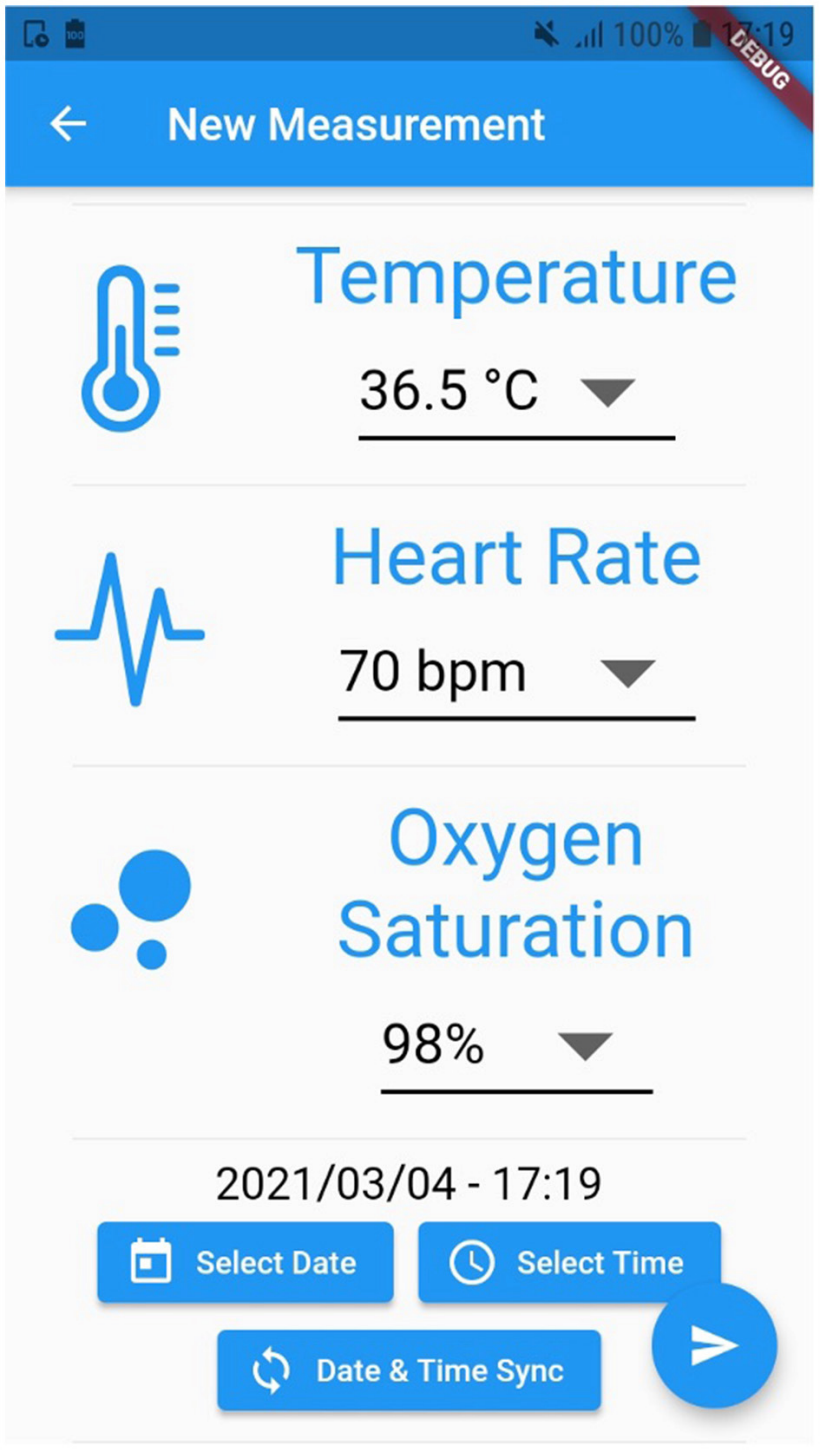
A possible instance of Step 2 of the *Patient*→*Medical Doctor* communication protocol.

**Figure 3:**
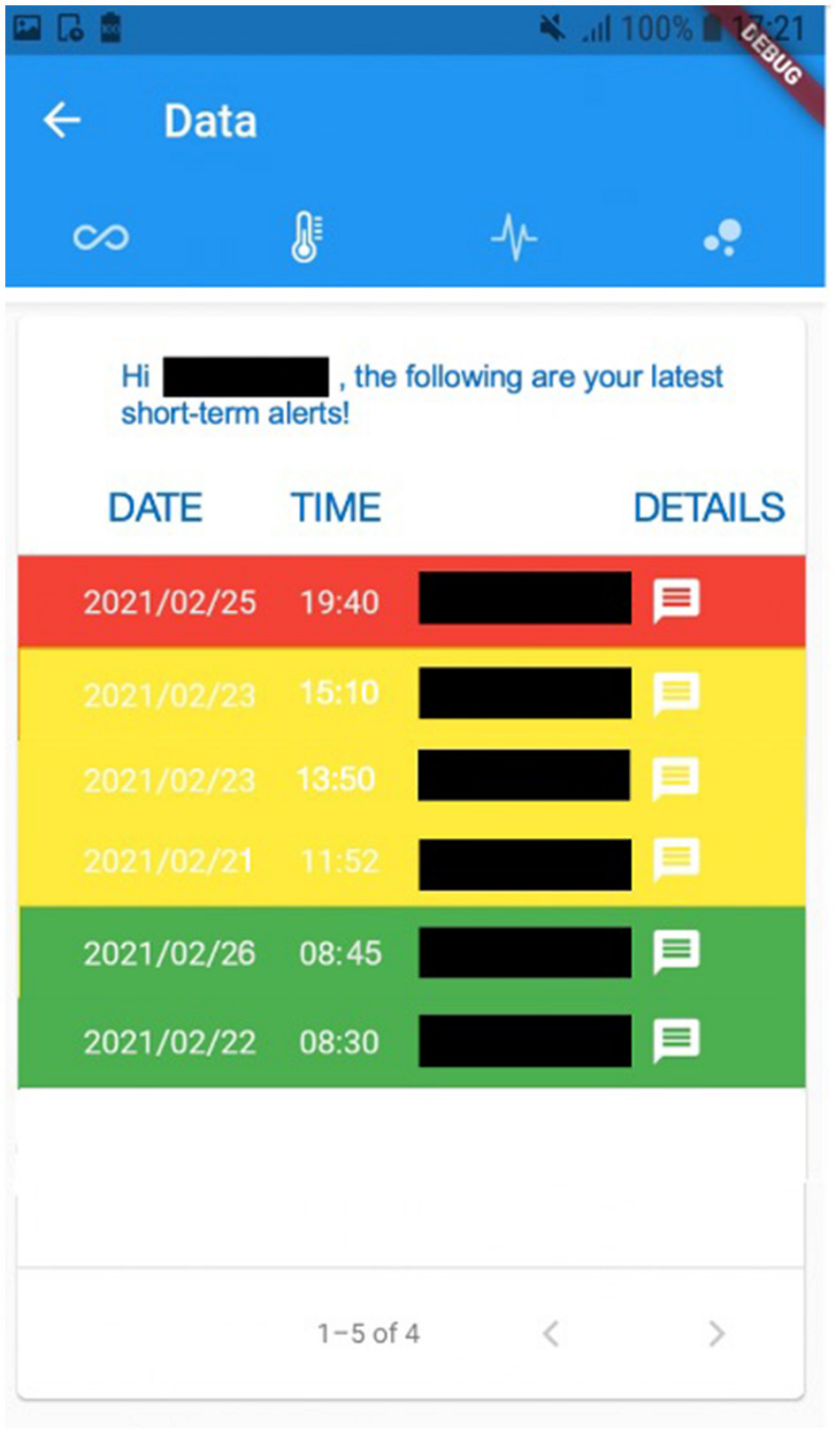
A possible instance of Step 4 of the *Patient*→*Medical Doctor* Communication Protocol, where the alerts are shown to the patient.

**Figure 4:**
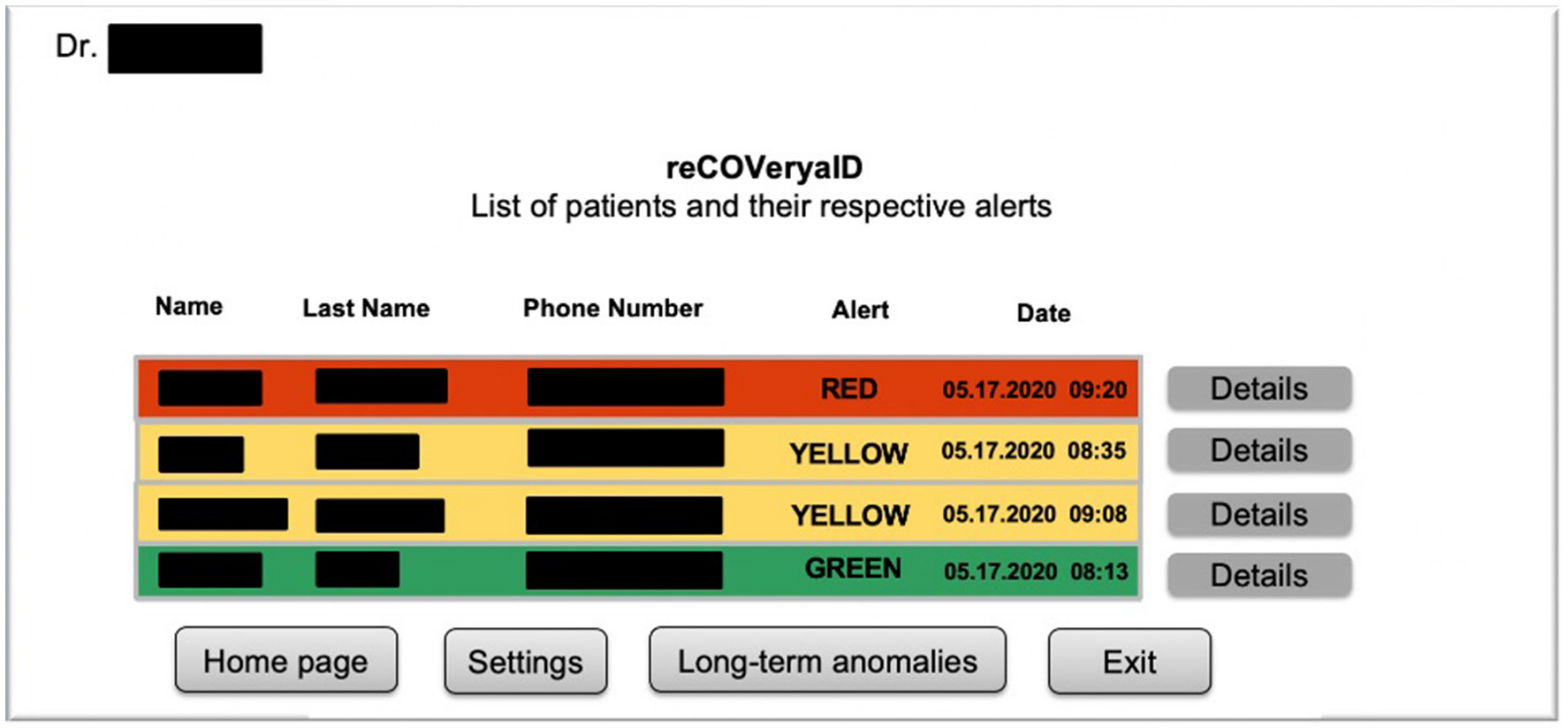
A possible instance of Step 6 of the *Patient*→*Medical Doctor* communication protocol.

For instance, Figure 2 exhibits a possible example of Step 2 of the Patient → Medical Doctor Communication Protocol; in this case, the current measurement is inserted by the patient manually via the app. A possible instance of Step 4 of the Communication Protocol depicted in Figure 1 is shown in Figure 3; in this case, the system shows the latest short-term alerts of a patient (the name is omitted for privacy reasons) ordered by descending timestamp. Additionally, Figure 4 shows one of the patient's alerts analyzed by the medical doctor, which combine the values of temperature, oxygen saturation, and heart rate. Then, the medical doctor can quickly click on the icon on the right to send a feedback message to the patient, who can easily receive it.

**Figure 5:**
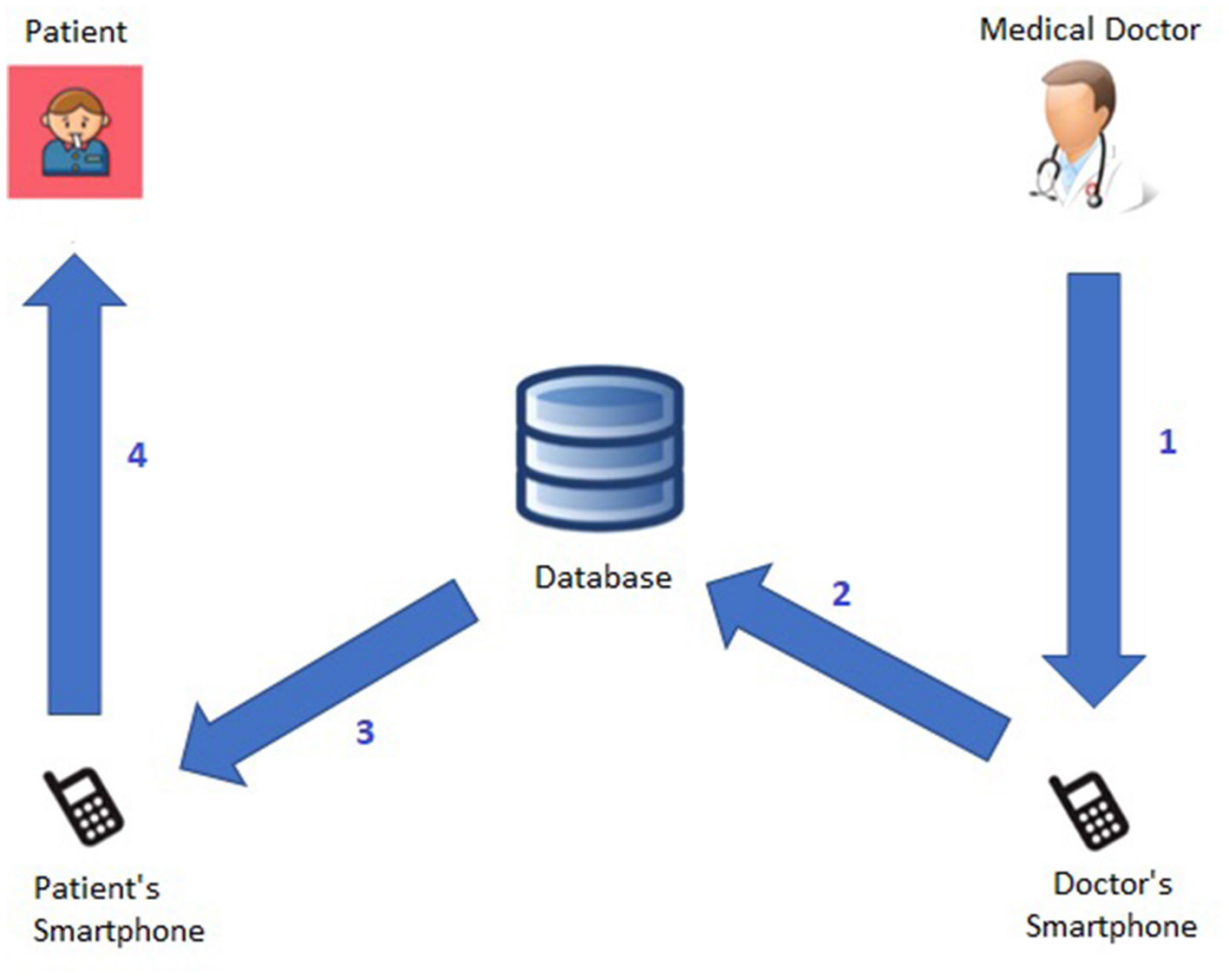
*Medical Doctor*→*Patient* communication protocol.

Symmetrically, Figure 5 shows the *Medical Doctor*→*Patient Communication Protocol*; specifically:

1. The medical doctor, after reviewing the patient's short-term and long-term alerts via the smartphone, sends the patient a feedback message.
2. The system stores the feedback message in the database.
3. The system forwards the feedback message to the patient's smartphone.
4. The patient has access to the medical doctor's feedback message.

The possible feedback messages are the following:

- OK, situation under control;
- Situation to be monitored more closely shortly;
- Red alert: call to the emergency service.

## 4. Knowledge-base rules

As also mentioned in Section 3, the system is intended to identify two categories of alerts:

- Alerts on the *last measurement*, which do not take into account the patient's history (see Section 4.1);
- Alerts on the *last* *N* *measurements*, generated after a more detailed analysis of the last *N* measurements of the patient (see Section 4.2).

The process of defining the rules for modeling the two categories of alerts was supported by medical doctors *Silvestro Volpe* and *Vittorio Palmieri*.

### 4.1. Short-term alerts

In order to design the rules to be adopted for generating alerts on the last measurement taken by the patient (which can be *red, yellow*, or *green*), we decided to use:

- Temperature threshold values *t*_*L*_, *t*_*H*_ ∈ ℝ, with *t*_*L*_ < *t*_*H*_;
- Oxygen saturation threshold values *OS*_*L*_, *OS*_*H*_ ∈ ℝ, with *OS*_*L*_ < *OS*_*H*_;
- Heart rate threshold values *HR*_*L*_, *HR*_*H*_, *HR*_*M*1_, *HR*_*M*2_ ∈ ℝ, with *HR*_*L*_ < *HR*_*M*1_ < *HR*_*M*2_ < *HR*_*H*_.

Hence, if *t*∈ℝ is the current temperature measurement, we have the following cases for the temperature:

- *t* > *t*_*H*_ → negative situation;
- *t*_*L*_ < *t* ≤ *t*_*H*_ → average situation;
- *t* ≤ *t*_*L*_ → positive situation.

Similarly, if *OS* ∈ ℝ is the current oxygen saturation measurement, we have the following cases for the oxygen saturation:

- *OS* < *OS*_*L*_ → negative situation;
- *OS*_*L*_ ≤ *OS* ≤ *OS*_*H*_ → average situation;
- *OS* > *OS*_*H*_ → positive situation.

Finally, if *HR*∈ℝ is the current heart rate measurement, we have the following cases for the heart rate:

- (*HR* < *HR*_*L*_)OR(*HR* > *HR*_*H*_) → negative situation;
- (*HR*_*L*_ ≤ *HR* < *HR*_*M*1_)OR(*HR*_*M*2_ < *HR* ≤ *HR*_*H*_) → average situation;
- *HR*_*M*1_ ≤ *HR* ≤ *HR*_*M*2_ → positive situation.

Therefore, initially considering only temperature and oxygen saturation values (which have been identified as the key parameters by medical doctors), after extensive discussion with medical doctors, we have obtained 9 possible combinations, corresponding to the following alert levels:

**Table:** 

*OS\t*	*t* ≤ *t_L_*	*t_L_* < *t* ≤ *t_H_*	*t_H_* < *t*
*OS_H_* < *OS*	green	yellow	yellow
*OS_L_* ≤ *OS* ≤ *OS_H_*	yellow	yellow	yellow
*OS* < *OS_L_*	red	red	red

Thus, the following rules are deduced for *red, yellow*, and *green* alerts:

- (*t* ≤ *t*_*L*_)AND(*OS*>*OS*_*H*_) → green alert;
- (*t*>*t*_*L*_AND*OS*≥*OS*_*H*_)OR(*OS*_*L*_ ≤ *OS* ≤ *OS*_*H*_) → yellow alert;
- *OS*<*OS*_*L*_ → red alert.

In addition, as validated by medical doctors, the inclusion of heart rate values (considered less important than the other parameters in any case) in the rules allows the set of green alerts to be narrowed, thus further extending the set of yellow alerts. Below are the rules for the identification of the alerts that also take heart rate into account.

- (*t* ≤ *t*_*L*_)AND(*OS*>*OS*_*H*_)AND(*HR*_*L*_ ≤ *HR* ≤ *HR*_*H*_) → green alert;
- (*t*>*t*_*L*_AND*OS*≥*OS*_*H*_)OR(*OS*_*L*_ ≤ *OS* ≤ *OS*_*H*_) OR (*t* ≤ *t*_*L*_AND*OS*>*OS*_*H*_AND(*HR*<*HR*_*L*_OR*HR*>*HR*_*H*_)) → yellow alert;
- *OS*<*OS*_*L*_ → red alert.

Such rules for finding alerts on the last measurement are stored within the specific knowledge base to be consulted before storing the new measurement in the database.

### 4.2. Long-term alerts

Unlike the *short-term alerts* discussed earlier, which depend only on the last measurement taken, the *long-term alerts* are generated by analyzing the patient's last *N* measurements, which are stored in the database.

In this subsection, we show two different versions of the rules for defining *long-term alerts*; the former are written as *triggers* directly in the database^5^, whereas the latter are even more restrictive in that, following medical doctors' guidelines, they are defined to identify even shorter negative trends that may be caused by COVID-19 variants.

#### 4.2.1. First version

As regards the first version, some specific triggers have been defined and implemented; such triggers are able to signal anomalies related to the last *N* measurements of *temperature, oxygen saturation* and *heart rate*. Specifically, in case *M* (*M* ∈ ℕ) measurements out of the last *N* (*M*<*N*) turn out to be “too close” to the red threshold of one of the three vital parameters, a long-term anomaly of the parameter concerned is signaled. Specifically, we apply the *Event-Condition-Action (ECA)* paradigm, exploited as follows;

- *EVENT*: after each insertion of a new measurement.
- *CONDITION*: when the parameter value (*temperature, oxygen saturation* or *heart rate*) in the last measurement is “too close” to the red threshold for that parameter.
- *ACTION*: the last *N* measurements of this parameter are analyzed; if at least *M* of these measurements are “too close” to the red threshold for this parameter, a *long-term anomaly* is generated.

In order to scientifically quantify the concept of “too close,” we define some additional thresholds allowing to identify *orange* alerts, i.e., yellow alerts close to the red ones. Here, we focus on the *SpO*2 analysis, but we made a similar assumption for *temperature* and *heart rate* as well.^6^ More specifically, we define a new threshold *SpO*2_*O*_ ∈ ℝ (where the *O* subscript stands for *Orange*), such that *SpO*2_*L*_<*SpO*2_*O*_<*SpO*2_*H*_; *SpO*2_*O*_ = *SpO*2_*L*_ + β · *SpO*2_*L*_, where β ∈ ℝ, β ∈ [0, 1] is the percentage of increase over *SpO*2_*L*_.

As a result, Algorithm 1 is triggered by every insert on the relational table of the database storing all the measurements (i.e., *measurements*); if the *SpO*2 value entered is within the orange zone (Algorithm 1), the *SpO2_Monitoring* function (Algorithm 2) is invoked.

**Algorithm 1:** SpO2_Anomaly trigger.

1: CREATE TRIGGER SpO2_Anomaly
AFTER INSERT ON measurements
FOR EACH ROW
WHEN (NEW.SpO2 ≤ *SpO*2_*O*_ and NEW.SpO2 > *SpO*2_*L*_)
EXECUTE PROCEDURE SpO2_Monitoring();

**Algorithm 2:** SpO2_Monitoring function.

**Input:** *M*, *N*
**Output:** *NEW*
1: CREATE OR REPLACE FUNCTION SpO2_Monitoring()
RETURNS TRIGGER AS $BODY$
2: DECLARE
3: inv_SpO2_number integer;
4: min_ts timestamp;
5: BEGIN
6: // Computing how many of the last N measurements
fall within the orange zone
SELECT count(*) INTO inv_SpO2_number
FROM measurements
WHERE measurements.SpO2 ≤ *SpO*2_*O*_ and
measurements.SpO2 > *SpO*2_*L*_ and id in (
SELECT id
FROM measurements
WHERE patient = NEW.patient
ORDER BY timestamp DESC LIMIT *N*);
7: // Looking for the minimum timestamp within the
last N measurements
SELECT min(timestamp) INTO min_ts
FROM measurements
WHERE id in (
SELECT id
FROM measurements
WHERE patient = NEW.patient
ORDER BY timestamp DESC LIMIT *N*);
// Checking whether to raise a SpO2 long-term alert
8: **if** (inv_SpO2_number ≥ *M*) **then**
9: INSERT INTO long_term_alerts
VALUES (NEW.patient, min_ts, now(), “Spo2
Long-Term Alert”);
10: **else**
11: NEW ← NULL;
12: **end if**
13: **return** *NEW*

Specifically, the *SpO2_Monitoring* function (Algorithm 2) accepts two arguments: the minimum number of short-term anomalies which raise a long-term alert (i.e., *M*), and the number of most recent short-term alerts to analyze (i.e., *N*); its output is the *NEW* object triggering Algorithm 1. In Lines 2–4 two variables (*inv_SpO2_number* and *min_ts*) are declared to hold the result sets of the queries, respectively, in Line 6 and Line 7. The query in Line 6 computes how many of the last *N* *SpO2* measurements fall in the orange zone, whereas the query in Line 7 retrieves the minimum timestamp within the last *N* measurements. Eventually, Lines 8–9 in Algorithm 2 generates a *SpO2 long-term alert* if at least *M* out of the last *N* SpO2 measurements are in the orange zone, by inserting long-term-alerts in the specific table.

#### 4.2.2. Second version

More recently, after further discussion with medical doctors, also due to the COVID-19 variants, we decided to make the algorithm even stricter. Specifically, with respect to the first version shown in Algorithms 1, 2, there are mainly three differences. First, in the new version we have expanded the set of possible measurements that can generate long-term alerts; in fact, Algorithm 3 in Supplementary material also adds red short-term alerts to the orange set (see Line 2). Second, while Algorithms 1, 2 just focus on measurements, independently from the dates, Algorithm 3 in Supplementary material just works on dates; as a result, if multiple measurements are taken on the same day, they are aggregated into a single value (i.e., the *average*), and consequently here *M* and *N* denote days, not measurements.^7^ Third, Algorithm 3 in Supplementary material further expands the set of short-term alerts that generate long-term alerts by giving more weight to recent history than to past history. This is obtained by defining and exploiting specific pattern scores (Line 20, Line 24, Line 28).

More specifically, Algorithm 3 in Supplementary material^8^ takes 5 arguments; (i) *measurements*- an array of recent measurements sorted from the most to the last recent; (ii) *M*- minimum number of anomalous measurements required to trigger a long-term alert; (iii) *N*- number of recent measurements to take into account; (iv) *span*- maximum number of days over which *M* anomalous measurements must be found to raise an alert; (v) *score_threshold*- the minimum pattern score which raises a long-term alert. The output is the *alerts* arraylist, which contains the long-term alerts generated by the last *N* measurements (of *oxygen saturation, temperature*, and *heart rate*) and triggered by the current one. Line 1 initializes (as empty) the arraylist holding the output, Line 2 defines the set of dangerous levels (in this case, *red* and *orange*), and Line 3 counts the number of measurements currently stored. Line 4 checks whether the values for *M* and *N* make sense (otherwise, the algorithm returns the error message *Incorrect values of the parameters*—Lines 58–59); in that case, if at least *M* measurements (Line 5) have already been stored (otherwise, the algorithm ends, since there are not enough records to compute long-term alerts—Lines 55–56), in case they are at least *N*, the *candidates* to be analyzed are *N* (Lines 6–7), otherwise they are something between *M* and *N* (Lines 8–9). Lines 11-12 respectively extract the start and the end date of the temporal interval to be analyzed, whereas Line 13 counts the number of days in the interval. In case (Line 14) the considered interval is within the range of the maximum number of days over which *M* anomalous measurements must be found to raise an alert (otherwise, the algorithm terminates, since the measurements are too sparse—Lines 52–53), the algorithm initializes to zero two counters for each vital (oxygen saturation, temperature, and heart rate); one for counting the short-term alerts falling in the *dangerous levels* set (Line 15), and one for counting the score achieved by the analyzed sequence of measurements (Line 16). Then, for each measurement (Line 17), the algorithm checks whether the short-term alert previously assigned to such a temperature measurement is within the set of dangerous levels (Line 18); in that case, the related counter is incremented (Line 19), and the pattern score is (linearly) incremented giving higher scores to recent history with respect to past history (Line 20). Lines 22–25 and Lines 26–29 do the same for, respectively, for oxygen saturation and heart rate candidates. Then, Lines 31–37 check if the analyzed temperature measurements raise a temperature long-term alert (symmetrically, Lines 38–44 and Lines 45–51 do the same for, respectively, oxygen saturation and heart rate); specifically, this happens if at least one of the following conditions occurs; either at least *M* measurements fall in the dangerous levels set, or the pattern score is at least equal to the *score_threshold* taken as fifth argument; in that case, a new alert object is initialized (Line 32)^9^, the alert type is set to *temperature* (Line 33), the start date (Line 34), and the end date (Line 35) are stored, then the object is pushed into the output arraylist (Line 36), which is eventually returned in Line 61.

Moreover, **Table 2** clarifies how Algorithm 3 extends the set of long-term alerts detected by Algorithm 1 (new patterns generating long-term alerts are highlighted in purple); specifically, the candidates pattern held in the first column is a string of length *N* from the most recent measurement to the last, where 0 means *no dangerous level*, and 1 means *dangerous level*. For instance, Table 1 shows an example of *SpO2* short-term alerts generating the 1101001 pattern (Table 2) for a generic patient.

**Table 1:** Example of *SpO2* short-term alerts generating the 1101001 pattern for a generic patient.

**Date**	**Short-term alert**	**Coding**
22-Nov-21	Red	1
23-Nov-21	Red	1
24-Nov-21	Yellow	0
25-Nov-21	Red	1
26-Nov-21	Green	0
27-Nov-21	Yellow	0
28-Nov-21	Red	1

**Table 2:** Long-term alerts (*M* = 5, *N* = 7, *score*_*threshold* = 18).

**Pattern (from the MOST to the LAST recent measurement)**	**Score**	**Does Algorithm 3 detect a long-term alert?**	**Does Algorithm 1 detect a long-term alert?**
0011111	15	YES	YES
0101111	16	YES	YES
0110111	17	YES	YES
0111011	18	YES	YES
0111100	18	YES	NO
0111101	19	YES	YES
0111110	20	YES	YES
0111111	21	YES	YES
1001111	17	YES	YES
1010111	18	YES	YES
1011010	18	YES	NO
1011011	19	YES	YES
1011100	19	YES	NO
1011101	20	YES	YES
1011110	21	YES	YES
1011111	22	YES	YES
1100110	18	YES	NO
1100111	19	YES	YES
1101001	18	YES	NO
1101010	19	YES	NO
1101011	20	YES	YES
1101100	20	YES	NO
1101101	21	YES	YES
1101110	22	YES	YES
1101111	23	YES	YES
1110000	18	YES	NO
1110001	19	YES	NO
1110010	20	YES	NO
1110011	21	YES	YES
1110100	21	YES	NO
1110101	22	YES	YES
1110110	23	YES	YES
1110111	24	YES	YES
1111000	22	YES	NO
1111001	23	YES	YES
1111010	24	YES	YES
1111011	25	YES	YES
1111100	25	YES	YES
1111101	26	YES	YES
1111110	27	YES	YES
1111111	28	YES	YES

Specifically, Table 2 refers to the following setting: *M* = 5, *N* = 7, *score*_*threshold* = *N*+(*N*−1) + (*N*−2) = 18; this is a reasonable scenario, since *N* covers a week, *M* is more than 70% of *N*, and *score*_*threshold* is sufficiently high to raise a long-term alert when a negative trend is identified in the most recent measurements, although less than *M* out of *N* short-term alerts fall within the dangerous levels set. In fact, Table 2 shows the different short-term alerts patterns for which either Algorithm 3 or Algorithm 1 raises a long-term alert^10^; the latter category includes all the scenarios where at least *M* measurements out of *N* are dangerous short-term alerts, whereas the former also includes those generating a long-term alert according to the additional condition (exceeded score threshold). For instance, one of the measurement patterns for which only Algorithm 3 in Supplementary material detects a long-term alert is 1110000, which occurs when the last 3 measurements are either *orange* or *red*; in this case, since less than *M* measurements are *orange* or *red*, Algorithm 1 does not raise a long-term alert; differently from it, Algorithm 3 (Lines 17–21, assuming they are temperature measurements) assigns it the score of 18 (7+6+5), which is equal to the chosen *score_threshold*, and thus generates a long-term alert (Line 31). As a result, Algorithm 3 in Supplementary material can quickly identify scenarios in which vital signs suddenly worsen.

## 5. Experimental evaluation

In this section we show the detailed experiments we conducted in order to validate the knowledge base rules defined in Section 4; specifically, in Section 5.1 we provide some details about the experimental setting and the data sets involving real patients provided by medical doctors. Then, Section 5.2 exhibits the evaluation of the rules for detecting short-term alerts, and Section 5.3 shows the detailed evaluation done to validate the rules to detect long-term alerts.

### 5.1. Experimental setting

After a long discussion with medical doctors, the following threshold values (Section 4) were chosen and used for the experimentation;

- *t*_*L*_ = 37; *t*_*O*_ = 37.5; *t*_*H*_ = 38.
- *SpO*2_*L*_ = 0.90; *SpO*2_*O*_ = 0.92; *SpO*2_*H*_ = 0.95.
- *HR*_*L*_ = 50; *HR*_*O*_1__ = 55; *HR*_*M*_1__ = 60; *HR*_*M*_2__ = 75; *HR*_*O*_2__ = 90; *HR*_*H*_ = 100.

We used two different data sets as the basis for our experiments. Both of them were kindly provided by the Pineta Grande Hospital in Caserta. The data sets are listed and quickly described below.

- *Hospital*, which includes anonymized data on 20 hospitalized patients infected with COVID-19. These data contain information on several temperature, oxygen saturation, and heart rate measurements over time, with at most one measurement per day.
- *USCA*,^11^ which stands for Special Continuity Care Units, includes anonymized data on 76 patients infected with COVID-19 in home telemonitoring. These clearly anonymized data contain information on several measurements of temperature, oxygen saturation, and heart rate over time, with at most one measurement per day.

### 5.2. Short-term alerts evaluation

#### 5.2.1. Goals

The main objective of evaluating short-term alerts is to measure the accuracy of the system against a ground truth provided by human annotators. More specifically, the goal is to quantify the ability of the rules defined in the knowledge base to identify red, yellow and green short-term alerts. In order to do so, we exploit the following metrics, whose formulas are then shown in detail in Section 5.2.2.

- *Average patient accuracy*, focusing on the average system performance on patients;
- *Global accuracy*, focusing on the system's performance in detecting short-term alerts, regardless of patients;
- *Precision*, focusing on the system's ability to avoid false positives when calculating short-term alerts. This is done for red, yellow, and green short-term alerts, respectively;
- *Recall*, focusing on the system's ability to avoid false negatives when calculating short-term alerts. This is done for red, yellow, and green short-term alerts, respectively.

#### 5.2.2. Methodology

In order to evaluate the accuracy of the rules discussed in Section 4, we defined a specific protocol, and we applied it both to the *Hospital* and to the *USCA* data set. The human annotators are medical doctors from the Pineta Grande Hospital in Caserta, who were not involved in the design process of the knowledge-base rules. Consequently, the annotators were informed about the short-term alerts (red, yellow, or green) with which they were to label each triple (temperature measurements, oxygen saturation, and heart rate), giving them only informal descriptions. So, they were asked to look at all triples of each patient and label them with the most appropriate color.

More formally, given a patient *j*, let $\left\{A_{ij}^{a}\right\}$_*i*∈[1,_*m*__*j*_]_ denote the set of short-term alerts returned by the algorithm in response to the *m*_*j*_ measurements of patient *j*. On the other hand, let $\left\{A_{ij}^{h}\right\}$_*i*∈[1,_*m*__*j*_]_ denote the set of short-term alerts associated by human annotators to the *m*_*j*_ measurements of patient *j*. Consequently, the accuracy of the knowledge-base rules for patient *j* is computed as follows (Equation 1):


(1) $$\begin{array}{l} patient_{j}accuracy=\frac{\left|{\left\{A_{ij}^{a}\right\}}_{i\in \left[1,m_{j}\right]}\cap {\left\{A_{ij}^{h}\right\}}_{i\in \left[1,m_{j}\right]}\right|}{m_{j}} \end{array}$$

Assuming to work on the data of *n* patients, the average accuracy per patient is computed as follows (Equation 2):


(2) $$average\;patient\;accuracy=\frac{\sum_{j=1}^{n}patient_{j}accuracy}{n}$$

After computing the *average accuracy*, it is straightforward to also calculate *variance* and *standard deviation* via the well-known formulas.

Additionally, assuming the total number of patients' measurements to be $\begin{array}{l} m=\sum_{j=1}^{n}m_{j} \end{array}$, the global system accuracy is defined as follows (Equation 3):


(3) $$\begin{array}{l} global\;accuracy=\frac{\left|{\left\{A_{ij}^{a}\right\}}_{i\in \left[1,m\right]}\cap {\left\{A_{ij}^{h}\right\}}_{i\in \left[1,m\right]}\right|}{m} \end{array}$$

So far, the mentioned metrics have been defined to compute the overall system accuracy. From now on, we focus on the system ability to compute red, yellow, and green alerts instead. Specifically, we do it by exploiting the classic precision (Equation 4) and recall (Equation 5) metrics.


(4) $$\begin{array}{l} precision_{x}=\;\frac{\left|{\left\{A_{ij}^{a}|A_{ij}^{a}=x\right\}}_{i\in [1,m]}\cap^{\text{​}}{\left\{A_{ij}^{h}|A_{ij}^{h}=x\right\}}_{i\in [1,m]}\right|}{\left|{\left\{A_{ij}^{a}|A_{ij}^{a}=x\right\}}_{i\in [1,m]}\right|}\\ \;x\;\in \{red,yellow,green\} \end{array}$$

(5) $$\begin{array}{l} recall_{x}=\;\frac{\left|{\left\{A_{ij}^{a}|A_{ij}^{a}=x\right\}}_{i\in [1,m]}\cap^{\text{​}}{\left\{A_{ij}^{h}|A_{ij}^{h}=x\right\}}_{i\in [1,m]}\right|}{\left|{\left\{A_{ij}^{h}|A_{ij}^{h}=x\right\}}_{i\in [1,m]}\right|}\\ \;x\;\in \{red,yellow,green\} \end{array}$$

#### 5.2.3. Results

As regards the *Hospital* data set, Table 3 summarizes its main information. In particular, we report the details about the number of analyzed patients (*n*), and the minimum, maximum, average, and total number of measurements per patient (respectively, *m*_*min*_, *m*_*max*_, *m*_*avg*_, *m*).

**Table 3:** Details of the *Hospital* data set.

**Parameter**	**Value**
*n*	20
*m*_*min*_	2
*m*_*max*_	13
*m*_*avg*_	6
*m*	120

**Figure 6:**
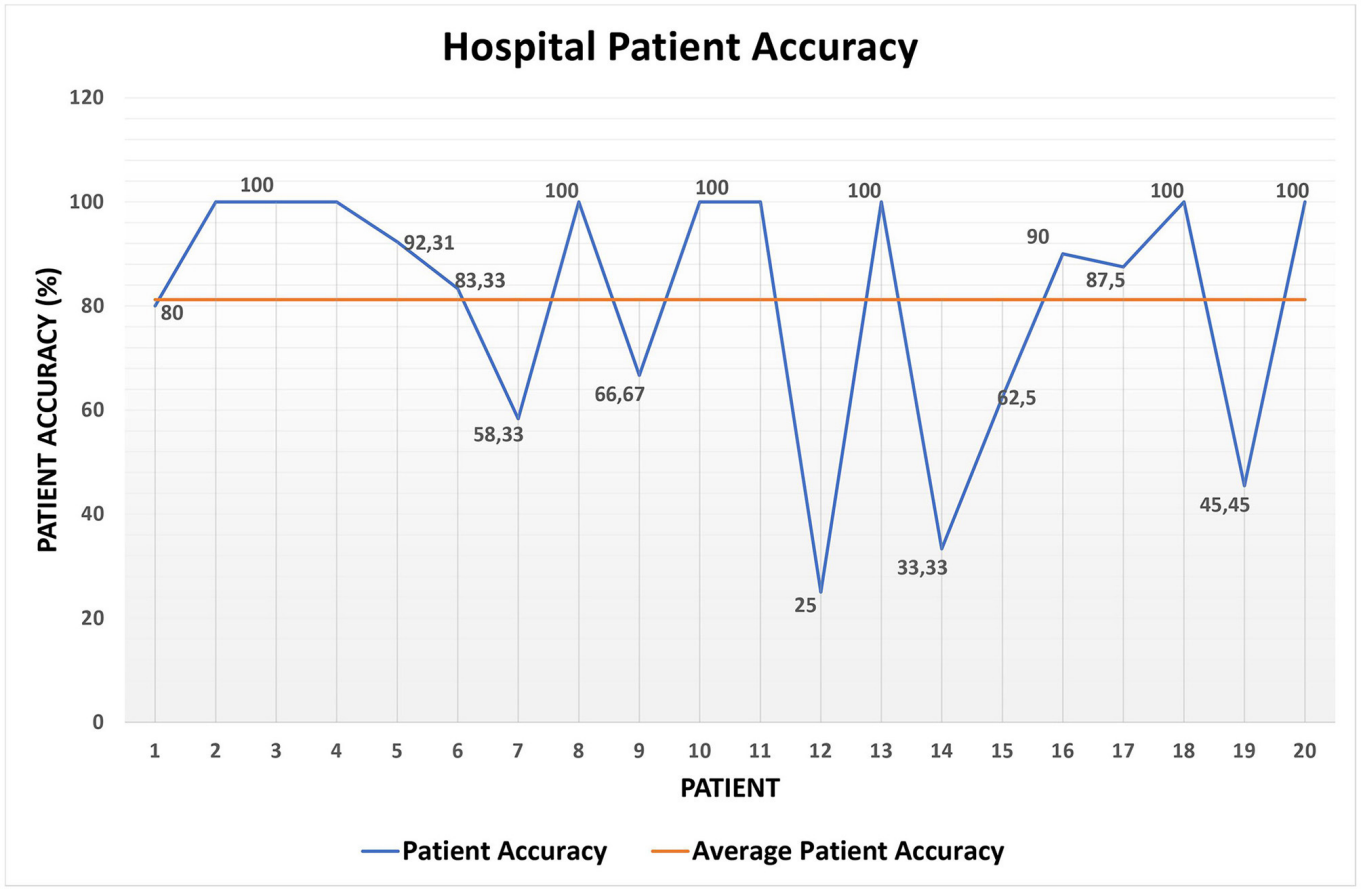
Patient accuracy on the *Hospital* data set.

Then, Table 4 shows the accuracy achieved by the system when computing short-term alerts on the *Hospital* data set. Additionally, Figure 6 exhibits in detail the *patient accuracy* values for each patient and the related *average patient accuracy*. Furthermore, Table 5 summarizes the results on the short-term alert precision and recall computed on the *Hospital* data set.

**Table 4:** Short-term alert accuracy on the *Hospital* data set.

**Metrics**	**Value**
Average patient accuracy	81.22%
Variance	0.06
Standard deviation	0.24
Global accuracy	77.50%

**Table 5:** Short-term alert Precision/Recall on the *Hospital* data set.

**x**	** $|{\left\{A_{ij}^{a}|A_{ij}^{a}=x\right\}}_{i\in \left[1,m\right]}$ **	** $\left|{\left\{A_{ij}^{a}|A_{ij}^{a}=x\right\}}_{i\in \left[1,m\right]}\right|$ **	**$\left|{\left\{A_{ij}^{h}|A_{ij}^{h}=x\right\}}_{i\in \left[1,m\right]}\right|$∩${\left\{A_{ij}^{h}|A_{ij}^{h}=x\right\}}_{i\in \left[1,m\right]}|$**	***precision*_*x*_**	***recall*_*x*_**
red	11	9	9	81.82%	100%
yellow	35	29	19	54.29%	65.52%
green	74	82	66	89.19%	80.49%

**Figure 7:**
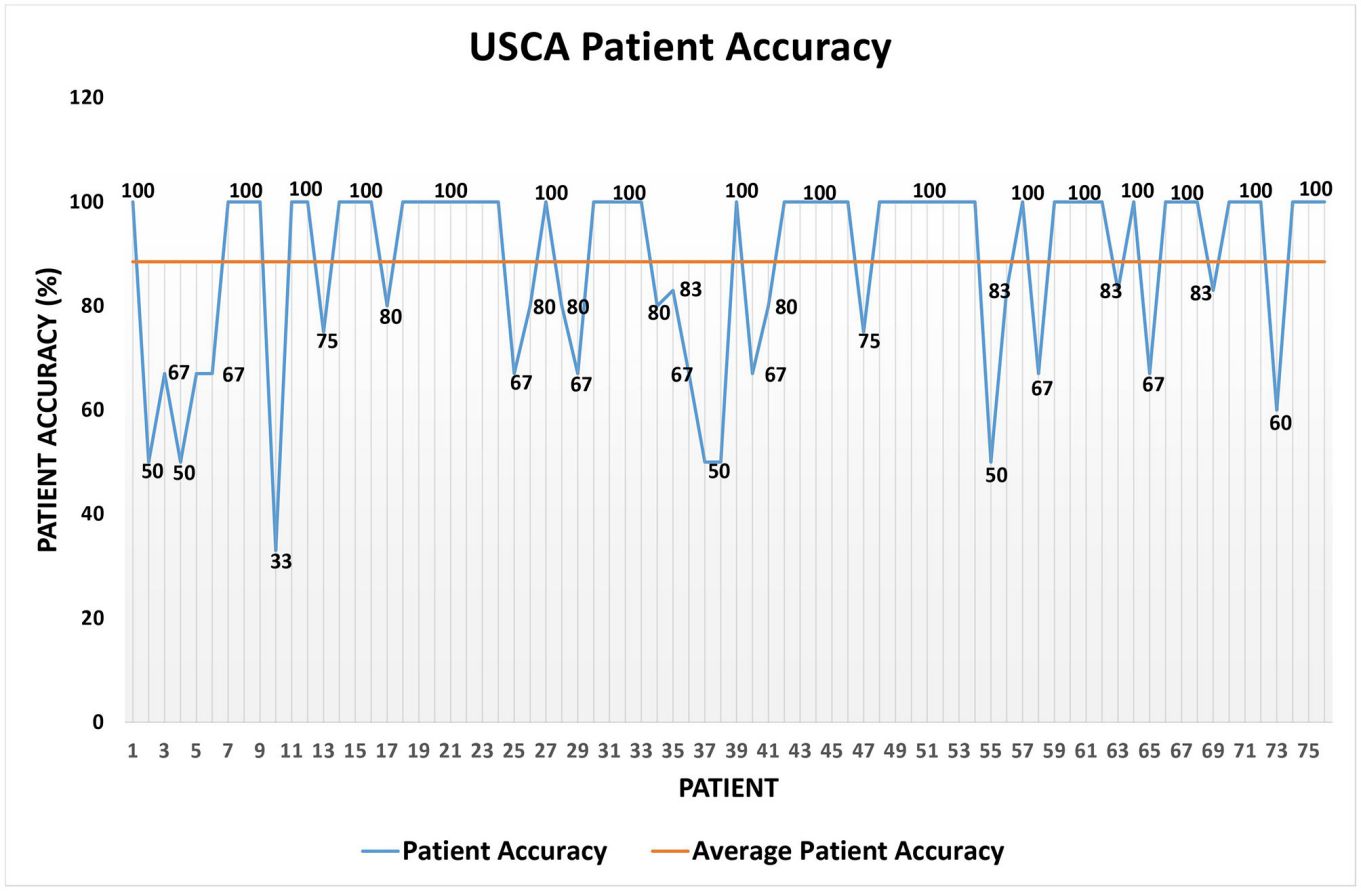
Patient accuracy on the *USCA* data set.

Regarding the *USCA* data set, Table 6 shows its details, Table 7 the patient accuracy, Figure 7 exhibits the patient accuracy detailed behavior, and Table 8 reports the precision/recall values.

**Table 6:** Details of the *USCA* data set.

**Parameter**	**Value**
*n*	76
*m*_*min*_	1
*m*_*max*_	6
*m*_*avg*_	3.76
*m*	286

**Table 7:** Short-term alert accuracy on the *USCA* data set.

**Metrics**	**Value**
Average patient accuracy	88.51%
Variance	0.03
Standard deviation	0.17
Global accuracy	86.36%

**Table 8:** Short-term alert Precision/Recall on the *USCA* data set.

**x**	** $|{\left\{A_{ij}^{a}|A_{ij}^{a}=x\right\}}_{i\in \left[1,m\right]}$ **	** $\left|{\left\{A_{ij}^{a}|A_{ij}^{a}=x\right\}}_{i\in \left[1,m\right]}\right|$ **	**$\left|{\left\{A_{ij}^{h}|A_{ij}^{h}=x\right\}}_{i\in \left[1,m\right]}\right|$∩${\left\{A_{ij}^{h}|A_{ij}^{h}=x\right\}}_{i\in \left[1,m\right]}|$**	***precision*_*x*_**	***recall*_*x*_**
red	26	26	26	100%	100%
yellow	110	72	72	65.45%	100%
green	150	188	150	100%	79.79%

#### 5.2.4. Discussion

Overall, the results shown in Table 4 and Figure 6 are promising, especially if we consider that they deal with the 120 measurements of the *Hospital* data set and that the system *global accuracy* value is not far from 80%.

Then, Table 5 demonstrates that the results shown in Table 4 and Figure 6 are even better than what we mentioned so far. This is due to at least three reasons; (i) *recall*_*red*_ = 100% implies that the algorithm for detecting red alerts did not miss any of them (i.e., there are no false negatives); (ii) the values of *precision*_*yellow*_ and *recall*_*yellow*_ are low, since the rules for detecting red alerts are, prudentially, even stricter than necessary, as confirmed by the value of *precision*_*red*_. However, in the middle of the pandemic we opted for a prudential choice; (iii) a remark similar to the one in (ii) can be made between yellow and green alerts; however, this case is much smaller than (ii), since the values of *precision*_*green*_ and *recall*_*green*_ are much higher than the ones of *precision*_*yellow*_ and *recall*_*yellow*_.

Regarding the *USCA*, a positive trend similar to the one highlighted on the *Hospital* data set, but on an even larger one (Table 6), is discovered. In fact, while the higher value of *average patient accuracy* in Table 7 with respect to the one in Table 4 can be biased by the lower value of *m*_*avg*_ (Tables 3, 6), the *global accuracy* in Table 7 sis unconditionally even higher than the one in Table 4. Additionally, the precision for the short-term yellow alerts is lower than the other values because the rules for detecting yellow alerts are, conservatively, even stricter than necessary; in fact, the algorithm often returned yellow alerts as a response to measurements that human annotators had labeled as green. This is why the system returned some false positives, but it was mainly because the testing was done during the spread of the delta variant of COVID-19, so the rules were tuned more tightly.

### 5.3. Long-term alerts evaluation

#### 5.3.1. Goals

The main objective of evaluating long-term alerts is to measure the accuracy of the system against a ground truth provided by human annotators. In this case, the goal is to quantify the ability of the rules defined in the knowledge base to identify long-term alerts via *Algorithm 1* and *Algorithm 3*. In order to do so, we exploit the following metrics, whose formulas are then shown in detail in Section 5.3.2.

- *Precision*, focusing on the system's ability to avoid false positives when calculating long-term alerts;
- *Recall*, focusing on the system's ability to avoid false negatives when calculating long-term alerts.

#### 5.3.2. Methodology

Similarly to what depicted for the short-term alerts, let us assume $\left\{LTA_{i}^{a}(t_{s})\right\}$_*i*∈[1,_*a*__*a*_]_ denotes the set of long-term alerts returned by the algorithm in response to the *m* measurements mentioned above. More specifically, let *a*_*a*_ denote the total number of long-term alerts returned by the algorithm, and let *t*_*s*_ be the timestamp when each of them is detected (clearly, being *t*_*s*_ the right endpoint of the analyzed long-term interval).

Symmetrically, let $\left\{LTA_{i}^{h}(t_{s})\right\}$_*i*∈[1,_*a*__*h*_]_ denote the set of long-term alerts associated by human annotators (i.e., by medical doctors) to the *m* measurements, where *a*_*h*_ is the total number of long-term alerts found by the annotators and *t*_*s*_ is still their timestamp. Clearly, human annotators were informed about the meaning of long-term alerts, but, differently from our algorithm, they could also have a look at different patient's symptoms and vitals.

Due to the not-too-large *m*_*avg*_ values for the *Hospital* (Table 3) and the *USCA* (Table 6) data sets, we opted for just computing global evaluations of *precision* (Equation 6) and *recall* (Equation 7).


(6) $$\begin{array}{l} \begin{array}{cc} precision & =\frac{\left|{\left\{LTA_{i}^{a}\left(t_{s}\right)\right\}}_{i\in \left[1,a_{a}\right]}\cap {\left\{LTA_{i}^{h}\left(t_{s}\right)\right\}}_{i\in \left[1,a_{h}\right]}\right|}{\left|{\left\{LTA_{i}^{a}\left(t_{s}\right)\right\}}_{i\in \left[1,a_{a}\right]}\right|} \end{array} \end{array}$$

(7) $$\begin{array}{l} \begin{array}{cc} recall & =\frac{\left|{\left\{LTA_{i}^{a}\left(t_{s}\right)\right\}}_{i\in \left[1,a_{a}\right]}\cap {\left\{LTA_{i}^{h}\left(t_{s}\right)\right\}}_{i\in \left[1,a_{h}\right]}\right|}{\left|{\left\{LTA_{i}^{h}\left(t_{s}\right)\right\}}_{i\in \left[1,a_{h}\right]}\right|} \end{array} \end{array}$$

Then, Table 9 exhibits the setting chosen for the algorithm for computing long-term alerts (Algorithm 3 in Supplementary material).

**Table 9:** Setting for the long-term alerts experiments.

**Parameter**	**Value**
*M*	2
*N*	4
*span*	7
*score*_*threshold*	4

#### 5.3.3. Results

Tables 10, 11 show the precision and recall values for, respectively, the *Hospital* and *USCA* data sets.

**Table 10:** Long-term alert Precision/Recall on the *Hospital* data set.

	**Precision (%)**	**Recall (%)**
Algorithm 1	60	60
Algorithm 3	55.56	100

**Table 11:** Long-term alert Precision/Recall on the *USCA* data set.

	**Precision (%)**	**Recall (%)**
Algorithm 1	80	40
Algorithm 3	75	90

#### 5.3.4. Discussion

Strictly speaking, the recall values achieved by Algorithm 3 are higher than the ones by Algorithm 1; this is mainly due to the main improvements we added to it, in order to broaden the set of short-term combinations generating long-term alerts (Section 4.2.2). Additionally, low values for precision (especially for the *Hospital* data set) are due to the fact that our algorithms were even stricter than medical doctors; however, the obtained precision values are just lower bounds, since the human annotators provided us with their ground truth before the delta-variant spread. For instance, when analyzing the ground truth, medical doctors often remove the long-term alert whenever a green short-term alert is detected after some red short-term alerts, whereas our algorithms are more cautious and keep the long-term alert. Furthermore, the achieved values are a bit biased by the small values for *m*_*avg*_, and by the fact that most of the short-term alerts in the data sets are not red.

## 6. Conclusion

In this paper, we have shown *reCOVeryaID*, an intelligent telemonitoring application for symptomatic, asymptomatic and pre-symptomatic coronavirus patients. More specifically, we described in detail the overall prototype, and verified the effectiveness of the knowledge-base rules for modeling and promptly detecting short-term and long-term alerts with data of real patients infected with COVID-19 and hospitalized at the Pineta Grande Hospital in Caserta. The high values of achieved accuracy demonstrate that a constant use of the *reCOVeryaID* framework by infected or potential patients can significantly help to promptly identify possible negative trends in their vitals, thus allowing an early hospitalization which can save his/her life.

Future work will be devoted to improve the current version of the developed prototype described in Section 3, in order to make it even more usable in a real scenario. Specifically, we intend to strengthen, with continuous interactions with the clinical staff involved in the COVID-19 emergency as well as the General Practitioners, the following features: - Acquisition of measurements, to be made also automatically via Bluetooth. This, in addition to facilitating data collection, will make it possible to guarantee safety and integrity of the inserted measurements. In addition, through this communication protocol, it will also be possible, if necessary, to hospitalize the patient, to interface the application with the different hospital systems, and also to allow the General Practitioners to continue to follow their patients, even if in the hospital; - Checking of the Tax Code, at the time of registration; - Allowing the patient to login by reading the health card or their digital identity; - Integrating the system for cases related to emergencies with the system of the First and Emergency Room, of the Operations Centres of Emergency Services, such as the Italian 118, Helicopter, etc.; -Integrating the system with the Hospital Information System (HIS), i.e., with the integrated set of IT tools used in healthcare to manage the administrative and clinical flows of a hospital, such as: Central Registry, Repository of Reports, Patient Management System (ADT), etc.; -Integrating the system with the healthcare platforms used by the General Practitioners.

Additionally, due to its simplicity and flexibility, *reCOVeryaID* is also easily transferable to other fields of application, not strictly related to the COVID emergency; in fact, it could be exploited to monitor patients with pathologies such as *diabetes* or *hypertension*, which expand its potential and further strengthen its acceptance by clinicians. Clearly, including other vital signs, such as blood pressure, in the system and then combining the different metrics in an original way, so as to detect early or even prevent potentially dangerous situations, involves further exploitation of knowledge representation and artificial intelligence techniques, such as logical inference.

## Data availability statement

The raw data supporting the conclusions of this article will be made available by the authors, without undue reservation.

## Ethics statement

Ethical approval was not required for the study involving human data in accordance with the local legislation and institutional requirements. Written informed consent to participate in this study was not required from the subjects in accordance with the national legislation and the institutional requirements.

## Author contributions

DD'A created and implemented the project. RR and AF provided significant help in experimenting on the two data sets. FA provided valuable advice and support as a biomedical engineer. DC is the project supervisor and provided his expertise in building knowledge bases. All authors contributed to the article and approved the submitted version.

## Footnotes

^1^The application and source code have not yet been released due to some constraints with the Italian Ministry. However, we sincerely hope to do so soon.

^2^ https://flutter.dev

^3^ https://dart.dev

^4^Automatic acquisition via Bluetooth is still in a prototype version due to constraints caused by the national grant funding to which the project is related. Anyway, it was tested on the following bluetooth pulse oximeter; https://www.nonin.com/products/3230/.

^5^Here, we show the code in *PL/pgSQL*.

^6^Due to space constraints, we only show the *PL/pgSQL* code to detect *oxygen saturation* long-term alerts.

^7^We developed additional code for this, which we do not report here.

^8^Its pseudocode is shown in Section 6.

^9^For the sake of clarity, we utilize an Object-Oriented-Programming-like notation.

^10^Table 2 clearly shows that the former category is a superset of the latter.

^11^From the Italian “Unità Speciali di Continuità Assistenziale.”

## References

[B1] Ali N. A. Khoja A. (2020). Telehealth: an important player during the COVID-19 pandemic. Ochsner J. 20, 113–114. 10.31486/toj.20.0039 32612460 PMC7310172

[B2] Bertoncelli C. M. Costantini S. Persia F. Bertoncelli D. D'Auria D. (2023). Predictmed-epilepsy: a multi-agent based system for epilepsy detection and prediction in neuropediatrics. Comput. Methods Prog. Biomed. 236, 107548. 10.1016/j.cmpb.2023.107548 37149974

[B3] Burdick H. Lam C. Mataraso S. Siefkas A. Braden G. Dellinger R. P.. (2020). Prediction of respiratory decompensation in Covid-19 patients using machine learning: the READY trial. Comput. Biol. Med. 124, 103949. 10.1016/j.compbiomed.2020.103949 32798922 PMC7410013

[B4] Caricchio R. Gallucci M. Dass C. Zhang X. Gallucci S. Fleece D.. (2021). Preliminary predictive criteria for COVID-19 cytokine storm. Ann. Rheumat. Dis. 80, 88–95. 10.1136/annrheumdis-2020-218323 32978237

[B5] Charles B. L. (2000). Telemedicine can lower costs and improve access. Healthcare Fin. Manage. 54, 66. 10915354

[B6] Chauhan V. Galwankar S. Arquilla B. Garg M. Di Somma S. El-Menyar A.. (2020). Novel coronavirus (COVID-19): leveraging telemedicine to optimize care while minimizing exposures and viral transmission. J. Emerg. Trauma Shock 13, 20–24. 10.4103/JETS.JETS_32_20 32308272 PMC7161346

[B7] Coupet S. Nicolas G. Louder C. Meyer M. (2021). When public health messages become stressful: managing chronic disease during COVID-19. Soc. Sci. Hum. Open 4, 100150. 10.1016/j.ssaho.2021.100150 33880443 PMC8030736

[B8] Danhieux K. Buffel V. Pairon A.. (2020). The impact of COVID-19 on chronic care according to providers: a qualitative study among primary care practices in Belgium. BMC Fam. Pract. 21, 255. 10.1186/s12875-020-01326-3 33278877 PMC7718831

[B9] D'Auria D. Moscato V. Postiglione M. Romito G. Sperli G. (2023). Improving graph embeddings via entity linking: a case study on Italian clinical notes. Intell. Syst. Appl. 17, 200161. 10.1016/j.iswa.2022.200161

[B10] De Lauretis L. Persia F. Costantini S. D'Auria D. (2023). How to leverage intelligent agents and complex event processing to improve patient monitoring. J. Logic Comput. 33, 900–935. 10.1093/logcom/exad016

[B11] Dimitroulas T. Bertsias G. (2020). Practical issues in managing systemic inflammatory disorders during the COVID-19 pandemic. Mediterranean J. Rheumatol. 31(Suppl. 2), 253–256. 10.31138/mjr.31.3.253 33196001 PMC7656127

[B12] Elliott M. Baird J. (2019). Pulse oximetry and the enduring neglect of respiratory rate assessment: a commentary on patient surveillance. Brit. J. Nurs. 28, 19. 10.12968/bjon.2019.28.19.1256 31680572

[B13] Gao Y. Cai G.-Y. Fang W. Li H.-Y. Wang S.-Y. Chen L.. (2020). Machine learning based early warning system enables accurate mortality risk prediction for COVID-19. Nat. Commun. 11, 5033. 10.1038/s41467-020-18684-2 33024092 PMC7538910

[B14] Hacker K. A. Briss P. A. Richardson L. Wright J. Petersen R. (2021). COVID-19 and chronic disease: the impact now and in the future. Prev. Chronic Dis. 18, E62. 10.5888/pcd18.210086 34138696 PMC8220960

[B15] Han Z. Wei B. Hong Y. Li T. Cong J. Zhu X.. (2020). Accurate screening of COVID-19 using attention-based deep 3D multiple instance learning. IEEE Trans. Med. Imaging 39, 2584–2594. 10.1109/TMI.2020.2996256 32730211

[B16] Hollander J. E. Carr B. G. (2020). Virtually perfect? Telemedicine for Covid-19. N. Engl. J. Med. 382, 1679–1681. 10.1056/NEJMp2003539 32160451

[B17] Ko S. Wang Z. Premkumar A. Qi T. Shuhua K. Lim Y, W.. (2023). Continuous vital signs monitoring in patients hospitalized at home: Burden or benefit? J. Am. Med. Direct. Assoc. 24, 547. 10.1016/j.jamda.2023.02.109 37011887 PMC10064244

[B18] Li W. Ma J. Shende N. Castaneda G. Chakladar J. Tsai J. C.. (2020). Using machine learning of clinical data to diagnose COVID-19: a systematic review and meta-analysis. BMC Med. Inform. Decis. Mak. 20, 247. 10.1186/s12911-020-01266-z 32993652 PMC7522928

[B19] Mason A. Rose E. Edwards C. J. (2020). Clinical management of Lupus patients during the COVID-19 pandemic. Lupus 29, 1661–1672. 10.1177/0961203320961848 33059530

[B20] Monaghesh E. Hajizadeh A. (2020). The role of telehealth during COVID-19 outbreak: a systematic review based on current evidence. BMC Public Health 20, 1193. 10.1186/s12889-020-09301-4 32738884 PMC7395209

[B21] Murali S. (2023). Machine learning and internet of things to improve patient health monitoring systems. Indian J. Nat. Sci. 14549, 56814–56820.

[B22] Persia F. Costantini S. Ferri C. Lauretis L. D. D'Auria D. (2021). “A smart framework for automatically analyzing electrocardiograms,” in 2021 Third International Conference on Transdisciplinary AI (TransAI) (IEEE), 64–67.

[B23] Scott D. A. McDougall R. (2017). The effective introduction of Lifebox pulse oximetry to Malawi. Anaesthesia 72, 675–677. 10.1111/anae.13897 28439874

[B24] Smith M. Withnall R. Blackadder-Coward J. Taylor N. (2021). Developing a multimodal biosensor for remote physiological monitoring. BMJ Mil. Health 169, 170–175. 10.1136/bmjmilitary-2020-001629 33542142 PMC10176328

[B25] Solé D. Komatsu M. K. Carvalho K. V. T. Naspitz C. K. (2009). Pulse oximetry in the evaluation of the severity of acute asthma and/or wheezing in children. J Asthma 36, 327–333. 10.3109/02770909909068225 10386496

[B26] Taguchi O. Hida W. Kikuchi Y. Miki H. Iijima H. Homma M.. (1994). Bronchial asthma and desaturation-assessment by pulse oximetry. Nihon Kyobu Shikkan Gakkai Zasshi 32, 115–120. 7602818

[B27] Takei R. Yamano Y. Kataoka K. Yokoyama T. Matsuda T. Kimura T.. (2020). Pulse oximetry saturation can predict prognosis of idiopathic pulmonary fibrosis. Respirat. Investig. 58, 190–195. 10.1016/j.resinv.2019.12.010 32160945

[B28] Totuk A. Bayramoglu B. Tayfur I. (2023). Reliability of smartphone measurements of peripheral oxygen saturation and heart rate in hypotensive patients measurement of vital signs with smartphones. Heliyon 9, e13145. 10.1016/j.heliyon.2023.e13145 36814605 PMC9939538

[B29] Wiffen L. Brown T. Brogaard Maczka A. Kapoor M. Pearce L. Chauhan M.. (2023). Measurement of vital signs by lifelight software in comparison to standard of care multisite development (VISION-MD): protocol for an observational study. JMIR Res. Protoc. 12, e41533. 10.2196/41533 36630158 PMC9878372

[B30] Wright A. Salazar A. Mirica M. Volk L. A. Schiff G. D. (2020). The invisible epidemic: neglected chronic disease management during COVID-19. J. Gen. Internal Med. 35, 2816–2817. 10.1007/s11606-020-06025-4 32666485 PMC7359916

