# Supplementary material for: An intelligent telemonitoring application for coronavirus patients: reCOVeryaID
Source: Front Big Data. 2023 Sep 18;6:1205766. doi: 10.3389/fdata.2023.1205766 (PMC10543687; doi:10.3389/fdata.2023.1205766)
Supplement: Supplementary file 1 [file Data_Sheet_1.pdf]

SUPPLEMENTAL MATERIALS

This sections contains the details of the Algorithm 3, described in section 4.

Table 1. Comments to Algorithm 3

Variable or Line(s)	Comment
$measurements$	an array of recent measurements sorted from the most to the last recent
M	minimum number of anomalous measurements required to trigger a long-term alert
N	number of recent measurements to take into account
$span$	maximum number of days over which M anomalous measurements must be found to raise an alert
$score\_threshold$	the minimum pattern score which raises a long-term alert
$alerts$	arraylist containing the long-term alerts generated by the last N measurements
4	checking correct values of the parameters
5	checking if there are enough stored values to proceed
$11 - 13$	computing the time interval to investigate
14	checking if the interval is included into the span of interest
17	for every measurement to be checked...
18	if the short-term temperature alert falls into a dangerous category...
19	the counter of short-term temperature alerts is incremented
20	the score for temperature alerts is updated
22	if the short-term SpO2 alert falls into a dangerous category...
23	the counter of short-term SpO2 alerts is incremented
24	the score for SpO2 alerts is updated
26	if the short-term heart-rate alert falls into a dangerous category...
27	the counter of short-term heart-rate alerts is incremented
28	the score for heart-rate alerts is updated
$31 - 36$	checking whether to raise a long-term alert for temperature
$38 - 43$	checking whether to raise a long-term alert for SpO2
$45 - 50$	checking whether to raise a long-term alert for heart rate

Algorithm 3 ComputeLongTermAlerts

Input: $measurements$, M, N, $span$, $score\_threshold$
Output: $alerts$

```
alerts ← [];
dangerous_levels ← {red, orange};
number_of_measurements ← count(measurements);
if (M > 0 AND N > 0 AND M ≤ N) then
    if (number_of_measurements ≥ M) then
        if (number_of_measurements ≥ N) then
            candidates ← N;
        else
            candidates ← number_of_measurements;
        end if
        start_date ← measurements[candidates-1].date();
        end_date ← measurements[0].date();
        interval ← (end_date - start_date).days();
        if (interval ≤ span) then
            count_temp, count_spo2, count_hr ← 0;
            temp_score, spo2_score, hr_score ← 0;
            for (i = 0 to candidates-1) do
                if (measurements[i].temp_Alert ∈ dangerous_levels) then
                    count_temp++;
                    temp_score = temp_score + candidates - i;
                end if
                if (measurements[i].spo2_Alert ∈ dangerous_levels) then
                    count_spo2++;
                    spo2_score = spo2_score + candidates - i;
                end if
                if (measurements[i].hr_Alert ∈ dangerous_levels) then
                    hr_temp++;
                    hr_score ← hr_score + candidates - i;
                end if
            end for
            if (count_temp ≥ M OR temp_score ≥ score_threshold) then
                long_term_alert = new LongTermAlert();
                long_term_alert.type ← temp;
                long_term_alert.start_date ← start_date;
                long_term_alert.end_date ← end_date;
                push(alerts,long_term_alert);
            end if
            if (count_spo2 ≥ M OR spo2_score ≥ score_threshold) then
                long_term_alert = new LongTermAlert();
                long_term_alert.type ← spo2;
                long_term_alert.start_date ← start_date;
                long_term_alert.end_date ← end_date;
                push(alerts,long_term_alert);
            end if
            if (count_hr ≥ M OR hr_score ≥ score_threshold) then
                long_term_alert = new LongTermAlert();
                long_term_alert.type ← hr;
                long_term_alert.start_date ← start_date;
                long_term_alert.end_date ← end_date;
                push(alerts,long_term_alert);
            end if
        else
            // Measurements are too sparse
        end if
    else
        // Not enough records to compute alerts
    end if
else
    // Incorrect values of the parameters
end if
return alerts
```
